# Transcription factor JUNB is required for transformation of EpCAM-positive hepatocellular carcinoma (HCC) cells into CD90-positive HCC cells in vitro

**DOI:** 10.1038/s41419-025-07602-3

**Published:** 2025-04-19

**Authors:** Yutaro Kawakami, Hikari Okada, Kouki Nio, Tomoyuki Hayashi, Akihiro Seki, Hidetoshi Nakagawa, Shinya Yamada, Noriho Iida, Tetsuro Shimakami, Hajime Takatori, Masao Honda, Shuichi Kaneko, Taro Yamashita

**Affiliations:** 1 Department of Gastroenterology, Kanazawa University Graduate School of Medical Science, Ishikawa, Japan; 2 Information-Based Medicine Development, Graduate School of Medical Sciences, Kanazawa University, Ishikawa, Japan

**Keywords:** Cell biology, Cancer stem cells, Metastasis

## Abstract

Hepatocellular carcinoma (HCC) harbors two types of stem cells—epithelial and mesenchymal stem cells. The mechanism by which epithelial EpCAM-positive HCC cells transform into mesenchymal CD90-positive HCC cells remains unclear. On peritumoral fibrotic nodules, epithelial HCC cells form communities with stromal cells, driving tumor growth and malignancy. We aimed to clarify the mechanism by which epithelial cell adhesion molecule (EpCAM)-positive HCC cells contribute to the phenotype of mesenchymal CD90-positive HCC cells that metastasize to distant sites by elucidating the interaction between EpCAM-positive HCC cells and fibroblasts. EpCAM-positive CD90-negative epithelial HCC cells (Huh1, Huh7, and HCC cells) were converted into metastasis-prone CD90-positive HCC cells by co-culture with fibroblasts (Lx-2 and Tig3-20). We identified the transcription factor JUNB as responsible for this altered phenotype. We found that the overexpression of JUNB in CD90-negative epithelial HCC cells resulted in significant transformation to mesenchymal CD90-positive HCC in vitro and in vivo, showing metastatic potential to the lungs. In addition, the JUNB expression in EpCAM-positive hepatoma cells was increased by paracrine stimulation with fibroblast-derived TGFb1. This study unravels the mechanism by which fibroblasts aggravate the malignancy of liver cancer, and the results suggest that JUNB may be a target for treating liver cancer metastasis.

## Introduction

Liver cancer is the third leading cause of death from malignant tumors worldwide, with hepatocellular carcinoma (HCC) accounting for 75–85% of primary liver cancers [1]. HCC has a poor prognosis owing to its drug resistance, high recurrence rate, and metastatic potential [2–5]. HCC recurrence occurs in 54% of patients after surgical resection, with recurrence rates of 35% and 70% within 1 year and 5 years. Extrahepatic metastasis occurs in 22% of recurrent cases and has been reported in autopsies in approximately 65% of cases of HCC-related deaths [6, 7].

Metastasis is a multistep process. Epithelial cancer cells lose intercellular adhesion and polarity, resulting in destruction of the basement membrane. These cells acquire motility and invasiveness in the tumor microenvironment (TME) and invade lymph nodes and blood vessels, forming metastatic lesions in the primary and other organs. The process is explained by epithelial–mesenchymal transition (EMT), which transforms epithelial cells into cells with mesenchymal properties [8, 9]. In addition, the TME around HCC induces angiogenesis, fibrosis, inflammation, and other effects related to carcinogenesis and growth, thereby promoting tumor growth and intra- and extrahepatic metastasis. TME comprises several stromal cell types, including lymphocytes, monocytes, stellate cells, fibroblasts, and vascular endothelial cells, which produce a variety of cytokines, chemokines, and growth factors, causing different physiological phenomena [10, 11]. Cancer-associated fibroblasts (CAFs) are among the TME constituents, present mainly in the marginal zone of tumors. They secrete various cytokines, such as HGF, TGFβ, VEGF, and PDGF, crosstalk with cancer cells, and induce tumor metastasis via EMT [12–15]. Tumor-associated macrophages (TAMs) and CAFs also create an immunosuppressive tumor environment that is favorable for tumor growth. Although this phenomenon is well understood, the transcription factors that determine the metastatic fate of epithelial cell adhesion molecule (EpCAM)-positive epithelial liver cancer cells remain largely unknown.

Cancer stem cells (CSCs) are implicated in the morphological heterogeneity of HCC, high recurrence rates, and resistance to anticancer drugs and radiation [16]. CSCs are progenitor cells with characteristics, such as self-renewal, limitless division, and heterogeneity. Owing to these characteristics, CSCs cause cancer symmetrically or asymmetrically, and because of their plasticity, they increase tumor malignancy while maintaining heterogeneity [17, 18]. We reported two types of CSCs in HCC—epithelial and mesenchymal. Epithelial CSCs express EpCAM as a cell surface marker, and are characterized by high serum alpha-fetoprotein (AFP) levels, venous invasion, tumor proliferation, enhanced invasiveness, and high resistance to anticancer drugs [16, 19]. On the contrary, mesenchymal CSCs express CD90 as a cell surface marker, and metastasize to the lungs and control distant metastasis. CD90-positive CSCs increase the motility of surrounding cancer cells by activating the TGFβ pathway [4]. CD90-positive CSCs are more drug sensitive than epithelial CSCs, and their extrahepatic metastasis is suppressed by anticancer drugs such as sorafenib [20]. EpCAM-positive CSCs self-renew and differentiate into EpCAM-positive and CD90-positive cancer cells; however, the transcription factors and signaling pathways involved in the plasticity of these CSCs remain unknown.

In this study, we investigated whether EpCAM CSCs transform into CD90 CSCs. We found that CAFs could transform the epithelial EpCAM CSCs into mesenchymal CD90 CSCs. On coculturing normal fibroblasts with EpCAM-positive human hepatoma cells, the latter were transformed into CD90-positive CSCs at high probability. We identified a transcription factor, JUNB, as the fate-determining factor that transforms epithelial CSCs to mesenchymal CSCs. By increasing the expression of JUNB in epithelial hepatoma cells, we could elucidate a part of the signaling pathway that renders the tumor environment conducive to the formation of CD90-positive CSCs.

## Results

### EpCAM-positive HCC cells are converted to CD90-positive cells when cocultured with fibroblasts

**Fig. 1:**
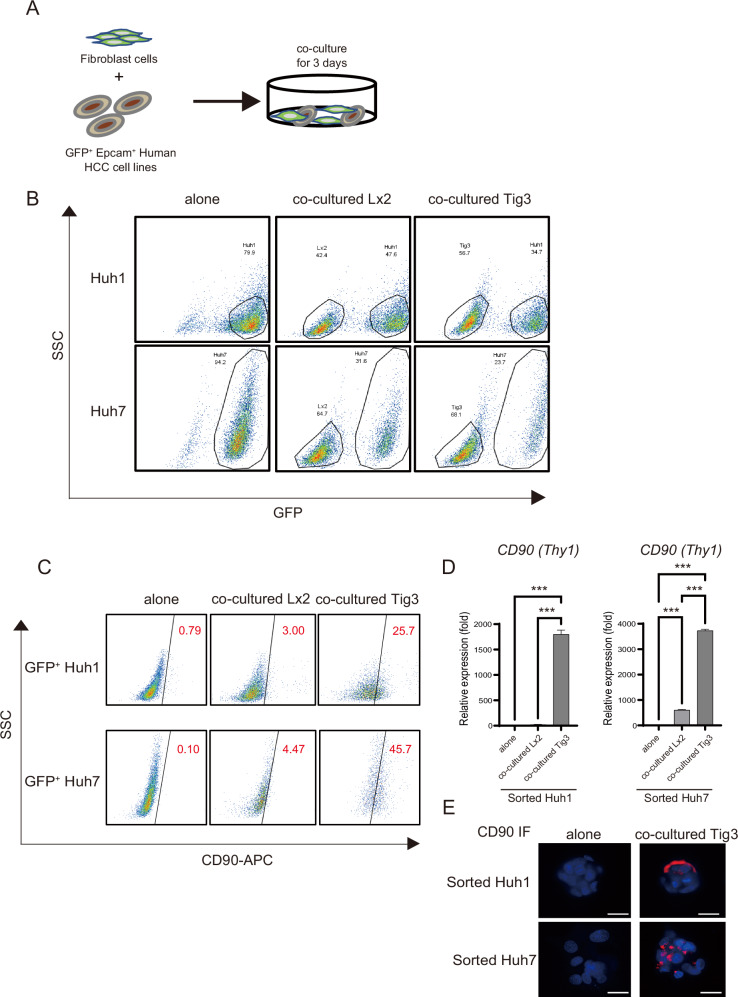
Coculture of EpCAM epithelial HCC cells with fibroblasts increases the expression of CD90 and the number of CD90-positive cancer cells in vitro. **A** The cell experiment schedule shows the coculture of GFP-labeled EpCAM-positive epithelial HCC cell lines Huh1 and Huh7 with fibroblasts Lx2 or Tig3-20 at a 1:2 ratio. **B**, **C** GFP-labeled HCC cells after coculture with fibroblasts were separated using GFP with flow cytometry (**B**), and the percentage of CD90-positive cells among GFP-positive HCC cells is shown (**C**). **D** Expression level of CD90 mRNA after separation of Huh1 and Huh7 cells cocultured with fibroblasts Lx2 or Tig3-20. **E** Expression of CD90 evaluated using fluorescent immunostaining after separation of Huh1 and Huh7 cells cocultured with fibroblasts Tig3-20. Data are presented as the mean (SD) (*N* = 3) and were analyzed using the one-way ANOVA. ^***^P < 0.001.

In highly malignant human liver cancer tissue, EpCAM- and CD90-positive cancer cells exist adjacent to each other. Nonmetastatic EpCAM-positive liver cancer cells transplanted into immunodeficient mice together with CD90-positive liver cancer cells metastasize to the lungs [4]. One of the reasons for the acquisition of metastatic potential in epithelial cancer is the transformation of EpCAM-positive cells into CD90-positive cells. However, the factor determining the transformation of CD90-negative cells to CD-positive ones is unknown. Therefore, we first evaluated whether EpCAM-positive liver cancer cells would transform into CD90-positive liver cancer cells upon coculture with hepatic stellate cells (HSC) Lx2 and lung fibroblasts TIG3-20 to obtain a higher metastatic potential than CD90-positive liver cancer cells. Stellate cells transform into hepatic fibroblasts, and cause liver fibrosis. We labeled the epithelial EpCAM-positive HCC cells with EGFP fluorescent tag to track changes in the cells, and directly mixed the cells and cultured them for three days to efficiently promote cell–cell interactions (Fig. 1A). When cocultured with Lx2 and TIG3-20 cells, the proliferation of epithelial EpCAM-positive HCC cells Huh1 and Huh7 was reduced by approximately 40% and 60% (Fig. 1B). Furthermore, Huh1 and Huh7 cells cultured with fibroblasts produced significantly more CD90-positive cells than the non-cocultured group. The conversion rate to CD90-positive cells was 0.79% for Huh1, 3.0% for Huh1 + Lx2, and 25.7% for Huh1 + Tig3-20, and 0.1% for Huh7, 4.47% for Huh7 + Lx2, and 45.7% for Huh7 + Tig3-20 (Fig. 1C). The conversion rate to CD90-positive cells was high in HCC cells after coculture with Tig3-20, and the CD90 mRNA levels were dramatically increased compared with those in the non-coculture group (Fig. 1D). Fluorescent immunostaining was used to observe the expression of CD90 on the surface of Huh1 and Huh7 cocultured with Tig3-20 (Fig. 1E). In Huh1 and Huh7, the expression of EpCAM tended to decrease after coculture with fibroblasts (Fig. S1A, B). Thus, EpCAM-positive HCC cells were efficiently transformed into CD90-positive HCC cells through cell-to-cell interactions with fibroblasts.

### EpCAM-positive HCC cells acquire metastatic potential when cotransplanted with fibroblasts in vivo

**Fig. 2:**
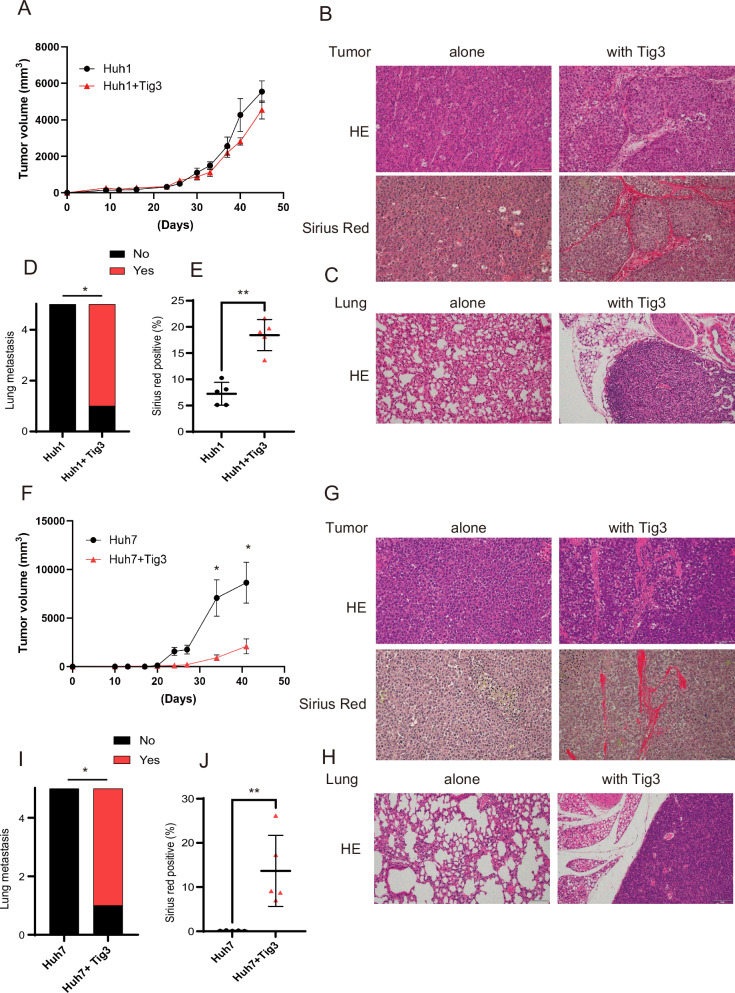
EpCAM-positive epithelial HCC cells metastasize at a high rate when mixed with fibroblasts and transplanted into immunodeficient mice. **A**–**E** Tumor growth curve (**A**), HE-stained images of subcutaneous tumors and Sirius red-stained images showing fibrosis (**B**), and HE-stained images of the lungs after subcutaneous implantation of a mixture of Huh1 and Tig3-20 in an immunodeficient model NOD-SCID mouse (*N* = 5) (**C**), probability of metastasis to the lungs (**D**), semi-quantified graph showing the degree of fibrosis in the tumor nodule of a subcutaneous tumor (**E**). **F**–**J** Tumor growth curve, HE-stained images of subcutaneous tumors, Sirius red-stained images, HE-stained images of the lungs after subcutaneous implantation of a mixture of Huh7 and Tig3-20 in a NOD-SCID mouse (*N* = 5), and probability of metastasis to the lungs. Semiquantified graph showing the degree of fibrosis in the nodules of a subcutaneous tumor. Data in **A**, **E**, **F**, and **J** are presented as mean (SD) (*N* = 5) and were analyzed using the Mann–Whitney *U*-test. ^*^P < 0.05. In **D** and **I**, data were analyzed using the *χ*^*2*^ test. ^***^P < 0.001.

Epithelial HCC cells were transformed into CD90-positive cells by coculturing with fibroblasts. However, it remained unclear whether CD90-positive HCC cells transformed by coculturing exhibit characteristics of mesenchymal CSCs. We, therefore, investigated whether the cells would acquire metastatic potential by subcutaneously inoculating epithelial HCC cells with equal amounts of Tig3-20 into immunodeficient mice. First, the growth curve of subcutaneous tumors in mice injected with Huh1 and Tig3-20 cells tended to decrease compared with that in mice in the single-implantation group (Fig. 2A). Pathologically, nodules were observed in the subcutaneous tumors, 47 days after coimplantation, possibly due to the coimplantation with fibroblasts (Fig. 2B). The coimplantation group showed a higher lung metastasis rate (80% in 4 out of 5 mice) than the single-implantation group (Fig. 2C, D). A similar study was also performed with Huh7, which has epithelial properties similar to those of Huh1. Tumor growth was significantly suppressed upon coimplantation of Huh7 and fibroblasts compared with that in the single-implantation group (Fig. 2F). Subcutaneous tumors coimplanted with Huh7 and Tig3-20 also showed nodules around the tumor (Fig. 2G). The coimplantation group also showed a significant increase in lung metastasis, with the metastasis rate being 80% in 4 out of 5 mice (Fig. 2H, I). Transplant recipients with fibroblastic peritumoral fibrous septa were more prone to lung metastases (Fig. 2E, J). These results clearly demonstrated that EpCAM HCC cells could metastasize to the lung when mixed with Tig3-20.

### Identification of cell fate-determining factors that mediate the conversion of epithelial HCC cells to CD90-positive cells by fibroblasts

**Fig. 3:**
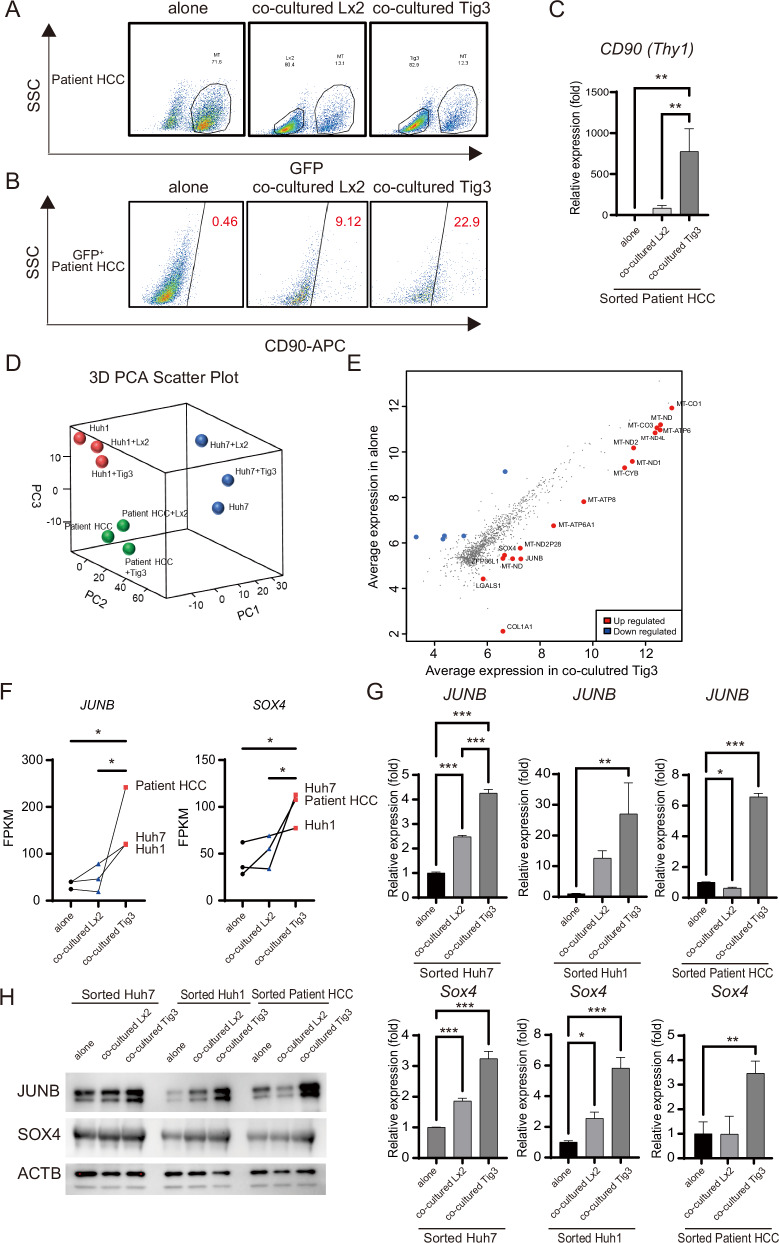
Induced of *SOX4* and *JUNB* in epithelial HCC cells by fibroblasts. **A**–**C** Human HCC cells isolated from surgical specimens were labeled with GFP and cocultured with fibroblasts. After coculture, cells were separated using GFP, and the percentage of CD90-positive cells is shown, along with the expression level of CD90 mRNA. **D**, **E** HCC cells Huh1, Huh7, and patient HCC were isolated with or without coculture with fibroblasts Lx-2 or Tig3-20. The isolated HCC cells were subjected to RNA-seq analysis, and the PCA diagram and scatter plot are shown. **F**–**H** mRNA levels of *JUNB* and *SOX4* assessed using RNA-seq, and mRNA and protein levels of JUNB and SOX4 in Huh1, Huh7, and patient HCC cocultured with fibroblasts. In **C**, **F**, and **G**, data are presented as the mean (SD) (*n* = 3) and were analyzed using the one-way ANOVA. ^*^P < 0.05, ^**^P < 0.01, ^***^P < 0.001. In **D**, data statistics were calculated using Huh1 (*N* = 1), Huh7 (*N* = 1), and patient HCC (*N* = 1) as three samples in total.

Because Huh1 and Huh7 are commonly used hepatoma cells, we investigated whether similar results would be obtained in clinical practice. We established EpCAM-positive, CD90-negative HCC cells from surgically resected human HCC tissue. The established patient HCC cells were labeled with EGFP and cocultured with Lx2 and Tig3-20. As with Huh1 and Huh7, coculture of patient HCC cells with fibroblasts suppressed their proliferation by ~60% (Fig. 3A). The CD90-positivity of patient HCC cells increased from 0.46% to 9.12% and 22.9% upon coculture with Lx2 and Tig3-20, respectively (Fig. 3B), but the EpCAM-positivity rate did not change (Fig. S1C). In addition, CD90 mRNA expression in patient HCC cells was significantly higher in the Tig3-20 coculture group (Fig. 3C).

Based on these results, we explored the effects of fibroblasts on epithelial HCC cells at the genetic level and the mechanism of transformation into CD90-positive cells. We performed RNA-seq analysis on Huh7, Huh1, and patient HCC cells cocultured with Lx-2 and Tig3-20. The PCA analysis of RNA-seq data showed that clusters in each HCC cell group even when cocultured with fibroblasts (Fig. 3D). This indicates that epithelial hepatoma cells do not change into completely different cells even when they acquire the ability to metastasize through fibroblasts. This was an interesting result, because the acquisition of metastatic capacity resulted in very few changes in cells at the genetic level. We simultaneously analyzed changes in the expression profiles of Huh7, Huh1, and patient HCC cells, comparing the coculture group with Tig3-20, which significantly converted from epithelial to CD90-positive mesenchymal cells, with the non-coculture group (Supplementary Table 1). The Tig3-20 coculture group showed a large number of mitochondria-related genes compared with the non-coculture group, and *JUNB*, *SOX4*, *COL1A1*, *LGALS1*, and *ZFP36L1* were detected with *P* < 0.05 or less (Fig. 3E). We checked if JUNB and SOX4 could be the genes determining the fate of CD90-positive cells. We confirmed that JUNB and SOX4 expression was significantly increased upon coculture with Tig3-20 (Fig. 3F–H). These results indicated that JUNB and SOX4 are required for CD90 positivity.

### Increased expression of JUNB in epithelial HCC cells transforms them into CD90-positive cells

**Fig. 4:**
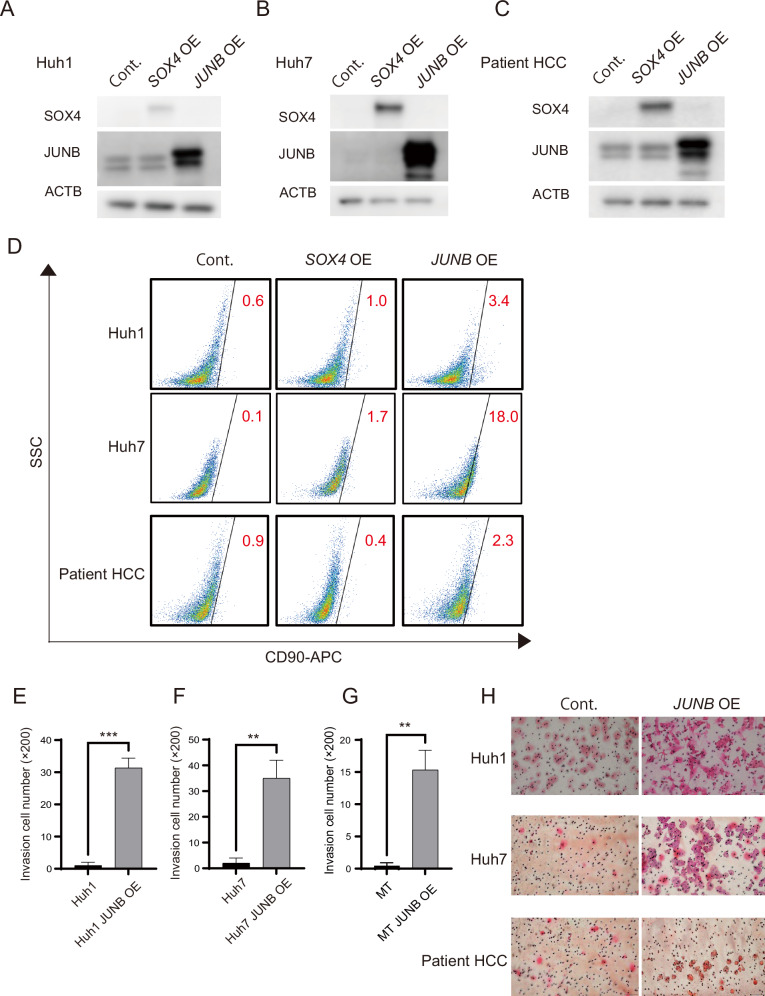
High JUNB expression in epithelial HCC cells transforms them into mesenchymal CD90-positive HCC cells in vitro. **A**–**C** Protein levels of SOX4 and JUNB after transfection of epithelial hepatoma cells Huh1 (**A**), Huh7 (**B**), and patient HCC (**C**). **D** Flow cytometry analysis shows the conversion rate of epithelial hepatoma cells Huh1, Huh7, and patient HCC transfected with SOX4 and JUNB to CD90-positive cells. **E**–**H** Graph of invasion rate and HE-stained images of membrane filters of epithelial hepatoma cells Huh1, Huh7, and patient HCC transfected with JUNB determined using invasion assay. In **E**–**G**, data are presented as the mean (SD) (*n* = 3) and were analyzed using the one-way ANOVA. ^**^P < 0.01, ^***^P < 0.001.

We examined whether overexpression of JUNB and SOX4 in epithelial HCC cells would transform them into CD90-positive cells with metastatic potential. SOX4 and JUNB were overexpressed in Huh1, Huh7, and patient HCC cells (Fig. 4A–C). SOX4-overexpressing cells did not reproducibly become CD90-positive compared to control cells. However, overexpression of JUNB rendered each cell type CD90-positive. The CD90-positivity of JUNB-overexpressing hepatoma cells increased from 0.6% to 3.4% for Huh1, from 0.1% to 18.0% for Huh7, and from 0.9% to 2.3% for patient HCC (Fig. 4D). The three types of EpCAM-positive hepatoma cells that overexpressed SOX4 showed no increase in CD90 expression. High JUNB expression in epithelial HCC significantly suppressed cell proliferation (Fig. S3A–C). This result was similar to that for coculture with fibroblasts. Next, we evaluated the invasive ability of EpCAM-positive cells to assess the acquisition of metastatic ability characteristic of CD90-positive cells, based on their transition to CD90-positive cells. The number of cells invading the Matrigel membrane containing extracellular matrix (ECM) components was significantly higher in the three JUNB-overexpressing HCC cell types than in the control cells (Fig. 4E–H). Immunostaining of subcutaneous tumors induced with Tig3-20 and hepatoma cells (Fig. 2B, F) revealed the presence of JUNB-positive cells around the tumor nodules (Fig. S2A, B). These results indicated that JUNB-positive epithelial HCC cells acquire invasive abilities and metastasize under the influence of fibroblasts.

### JUNB-overexpressing epithelial liver cancer has a high probability of metastasizing to the lungs

**Fig. 5:**
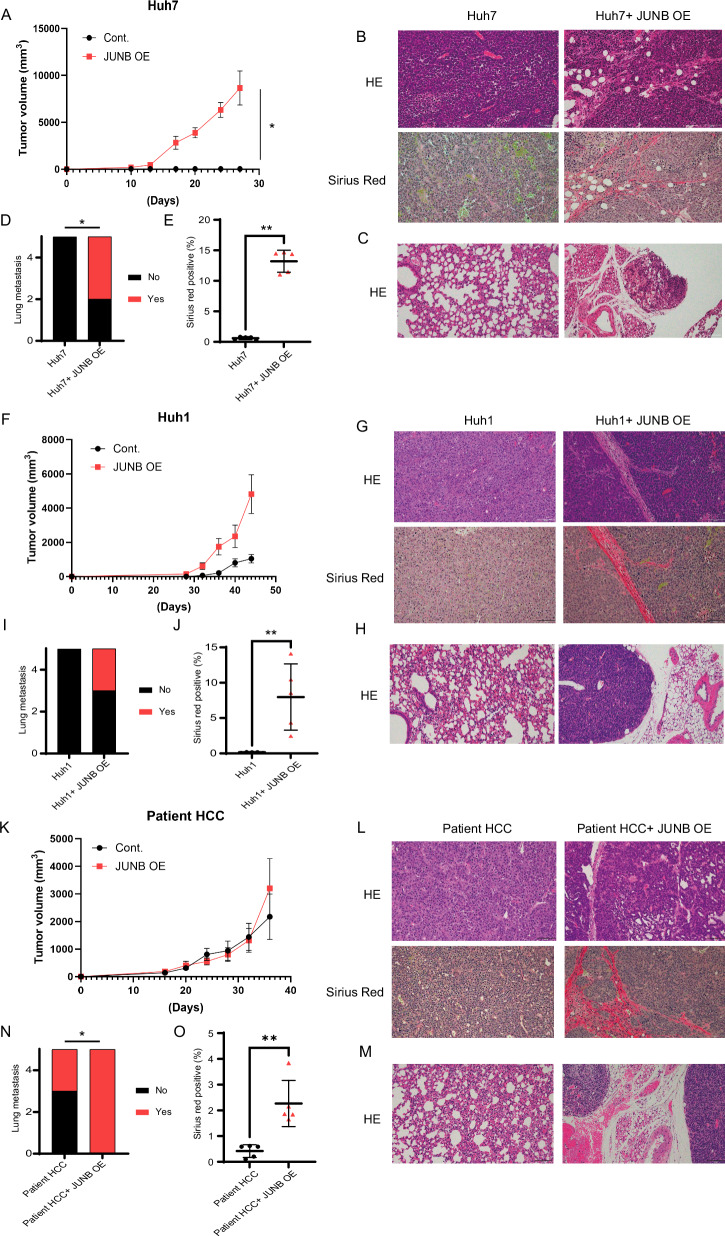
JUNB overexpression in epithelial HCC cells induces lung metastasis in vivo. **A**–**E** JUNB-overexpressing Huh1 cells were subcutaneously transplanted into an immunodeficient NOD-SCID mouse model (*n* = 5). The tumor growth curve after transplantation, HE-stained images of the subcutaneous tumor, Sirius red-stained image, positivity rate for fibrosis, the probability of metastasis to the lungs, and HE-stained images of the lungs are shown. **F**–**J** The tumor growth curve after subcutaneous transplantation of JUNB-overexpressing Huh7 into NOD-SCID mice (*n* = 5). HE-stained images of subcutaneous tumor, Sirius Red stain image, and positivity rate for fibrosis, probability of metastasis to the lungs, and HE-stained images of the lungs are shown. **K**–**O** Tumor growth curve after subcutaneous transplantation of JUNB-overexpressing patient HCC into NOD-SCID mice (*n* = 5), HE-stained images of the subcutaneous tumor, Sirius Red stain image and positivity rate for fibrosis, probability of metastasis to the lungs, and HE-stained images of the lungs are shown. **A**, **E**, **F**, **J**, **K**, and **O**, data are presented as the mean (SD) (*n* = 5) and were analyzed using the Mann–Whitney *U*-test. ^*^P < 0.05, ^*^P < 0.01. In **D**, **H**, and **L**, data are analyzed using the *χ*^*2*^ test. ^*^P < 0.05.

Having identified the EMT and signal pathway regulated by JUNB, we investigated whether JUNB actually promotes distant metastasis in vivo. To verify whether JUNB-overexpressing Huh7, Huh1, or patient HCC cells transform into CD90-positive HCC cells, we subcutaneously implanted them into NOD-SCID immunodeficient mice and observed tumor progression. Compared to the tumor formation curves obtained using coculture with fibroblasts, the tumor formation using JUNB-overexpressing Huh7 cells was significantly promoted compared with that in the control (Fig. 5A). Histological analysis of subcutaneous tumors revealed fibrous septa around the tumor because of JUNB overexpression (Fig. 5B). Compared with control cells, JUNB-overexpressing Huh7 cells significantly increased the incidence of lung metastasis, which increased from 0% (0 of 5 mice) to 60% (3 of 5 mice) (Fig. 5C, D). The progression of fibrosis around the tumor nodule was increased by the overexpression of JUNB, which increased the positivity of Sirius Red (Fig. 5E). The lung metastatic potential of Huh1 cells was also evaluated in NOD-SCID immunodeficient mice. JUNB-overexpressing Huh1 cells also enhanced the growth of subcutaneous tumors (Fig. 5F). Moreover, JUNB-overexpressing Huh1 subcutaneous tumors showed peritumoral fibrous thickening (Fig. 5G, J). JUNB overexpression in Huh1 cells increased the probability of lung metastasis from 0% (0 of 5 mice) to 40% (2 of 5 mice) (Fig. 5H, I). The results obtained with the cell line were similar to those obtained with patient HCC, and JUNB overexpression induced lung metastasis from 40% (2 of 5 mice) to 100% (5 of 5 mice) (Fig. 5K–O).

These results indicate that increased JUNB expression contributes to the metastasis of human epithelial HCC cells to other organs. Similar results were obtained in vivo, with JUNB activating the ECM tissue signaling pathway to induce peritumoral fibrosis.

### Analysis of signal pathways induced by increased JUNB expression in epithelial liver cancer cells

**Fig. 6:**
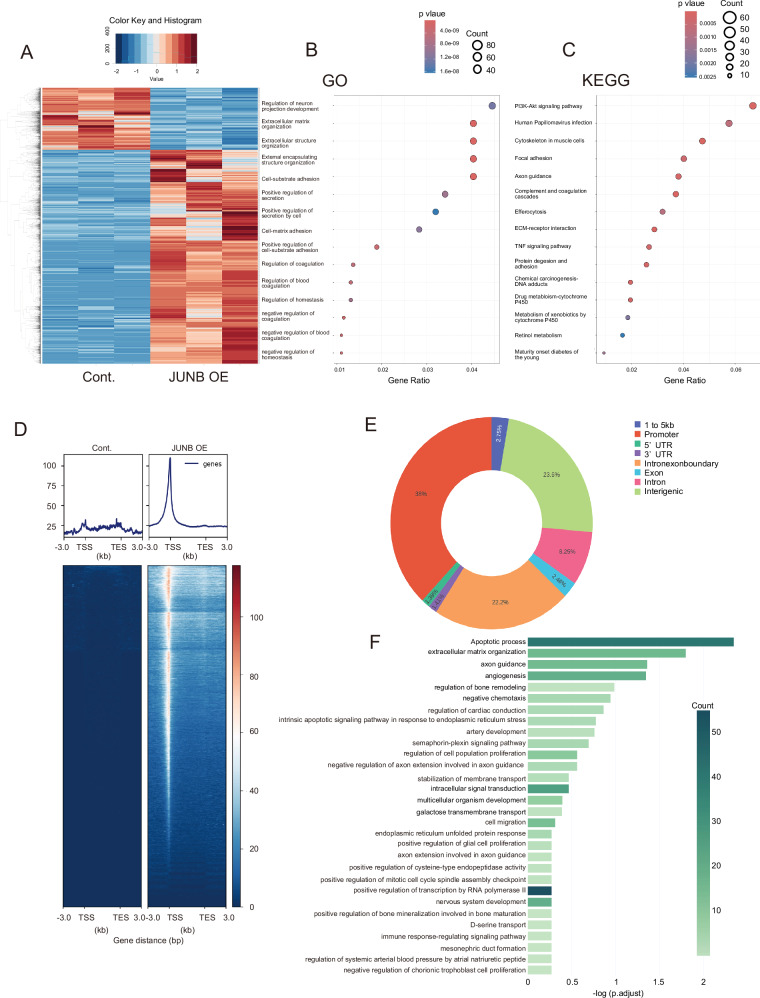
Analysis of gene expression and pathways directly altered in JUNB-transfected epithelial hepatoma cells. **A**–**C** Heat map of 2957 genes with *P* < 0.05 identified using RNA-seq analysis of JUNB-overexpressing Huh1 (*n* = 3), GO enrichment analysis, and KEGG pathway analysis. **D**–**F** Diagram showing the increased JUNB-binding peak due to overexpression of JUNB, distribution diagram of JUNB-binding to gDNA (*n* = 1), and graph depicting the results of GO analysis of genes bound to gDNA based on integrating RNA-seq and ChIP-seq data.

Next, we explored the phenomenon associated with JUNB overexpression in epithelial hepatoma cells, and its role in the acquisition of metastatic potential. We first assessed the mRNA levels of EMT-related genes *TGFb1*, *TWIST2*, *ZEB1*, *ZEB2*, *SNAIL1*, and *SLUG*, which promote metastasis, using RT-PCR. Increased JUNB expression in Huh1 and Huh7 significantly promoted the expression of EMT-related genes (Fig. S4A, B). In JUNB-overexpressing patient HCC cells, the expression of *TGFb1*, *ZEB2*, and *SLUG* was significantly higher than in control cells (Fig. S4C). Although JUNB promoted the expression of EMT-related genes, we did not know whether it directly controls the EMT. Next, we performed RNA-seq analysis of mRNA from control and JUNB-overexpressing cells to predict JUNB-induced changes in physiological phenomena other than EMT. We picked 2957 genes whose expression levels were significantly different (*P* < 0.05 or less) and prepared a heat map (Fig. 6A) (Supplementary Table 2). Among the significantly modulated genes, we selected only the ones significantly upregulated by JUNB and performed gene ontology (GO) analysis. Interestingly, gene sets enriched by JUNB related to ECM organization and cell–matrix adhesion rose to the top, with *P* < 0.001 or less (Fig. 6B). Furthermore, Kyoto encyclopedia of genes and genomes (KEGG) analysis for JUNB-upregulated genes, with *P* ≤ 0.001, revealed enrichment of the PI3K-Akt signaling pathway related to cell proliferation, cytoskeleton in muscle cells, ECM–receptor interaction, and focal adhesion related to ECM deposition (Fig. 6C). These results confirmed that in hepatocytes JUNB performs life and survival functions via activation of the AKT signaling pathway, and that it is involved in the production of fibrous components such as ECM. After deciphering the changes in gene expression and physiological phenomena caused by increased JUNB expression in epithelial HCC cells, we performed ChIP-seq using an antibody against JUNB, to identify the JUNB-binding regions on the genome. The overexpressed JUNB could bind to 11,928 genomic regions in epithelial HCC cells (Fig. 6D). A graph showing the 11,928 JUNB binding regions, broken down into smaller regions, is shown in Fig. 6E. Of these, JUNB could most frequently bind to promoter regions (38%), followed by intergenic (23.6%), intron–exon boundary (22.2%), and intron (8.25%) regions. After identifying the genome-binding sites of JUNB in epithelial HCC cells, we analyzed these results together with the data for 2957 genes whose expression was significantly altered in RNA-seq analysis to explore the JUNB signaling pathway. Our results indicated that JUNB transforms epithelial HCC cells into mesenchymal cells by significantly upregulating the signaling pathways related to apoptosis, ECM organization, and angiogenesis (Fig. 6F).

### Treatment of JUNB-overexpressing epithelial liver cancer cells with recombinant TGFb1 enhances the rate of conversion to CD90-positive cells

**Fig. 7:**
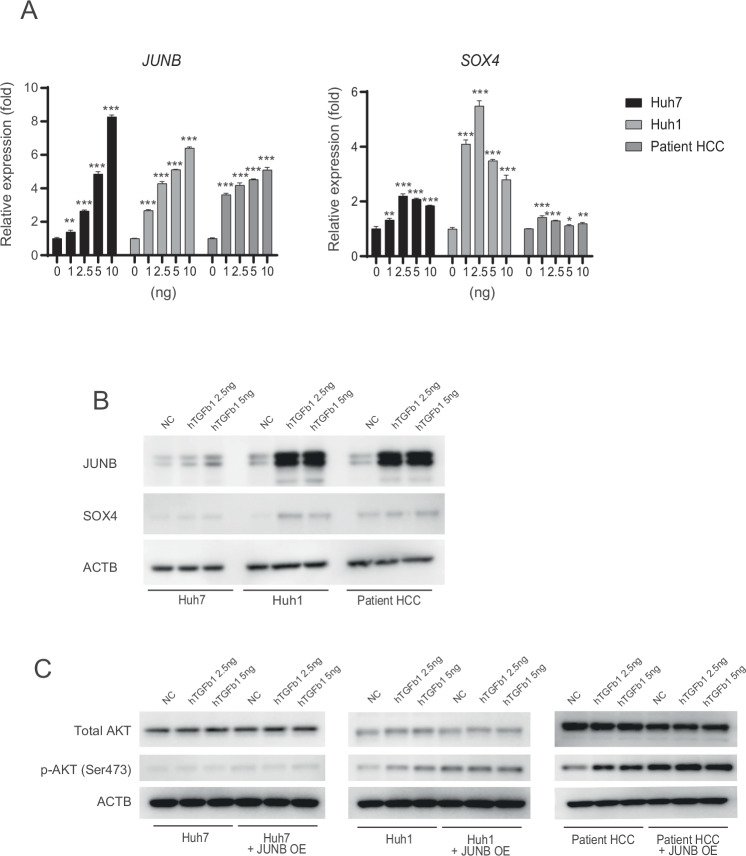
Stimulation with TGFb1 increases the expression of JUNB in epithelial HCC cells and promotes the rate of JUNB-mediated transformation into CD90-positive cells. **A**, **B** mRNA and protein levels of JUNB and SOX4, induced in a concentration-dependent manner, 24 h after the addition of TGFb1 to the epithelial HCC cell lines Huh7, Huh1, and patient HCC. **C** Activation of total AKT/pAKT (Ser473), total SMAD3/pSMAD3 (Ser423/425), and total JNK/pJNK (Thr183/Tyl185), 24 h after the addition of 2.5 ng and 5 ng of TGFb1 recombinant protein to the epithelial HCC cell lines Huh7, Huh1, and patient HCC overexpressing JUNB. In A, data are presented as the mean (SD) (*n* = 3) and were analyzed using the one-way ANOVA. ^*^P < 0.05, ^**^P < 0.01, ^***^P < 0.001.

Although JUNB is often reported to be involved in apoptosis, acting as AP1, and is a cell death signal, increased JUNB expression promoted tumor growth in vivo (Fig. 5A, E, and I). The induction of cell proliferation signal by JUNB may be due to the activation of AKT signaling (Fig. 6C). However, the upstream pathway that increases the expression of JUNB in epithelial hepatoma cells remains unknown. Mesenchymal CD90-positive hepatoma cells have been suggested to be produced by TGFb1 treatment [4]. We therefore treated three types of CD90-negative epithelial hepatoma cells with purified TGFb1 protein, JUNB expression significantly increased in a TGFb1 concentration-dependent manner (Fig. 7A, B), and the conversion rate to CD90-positive cells increased to 1.7% in Huh1, 2.75% in Huh7, and 2.79% in patient HCC (Fig. S5A–C). On the contrary, SOX4 expression was not increased in a TGFb1 concentration-dependent manner. The conversion rate of epithelial hepatoma cells with high JUNB expression to CD90 positive cells upon treatment with purified TGFb1 was significantly increased compared with that in untreated cells, being 7.51% for Huh1, 10.5% for Huh7, and 10.7% for patient HCC (Fig. S5A–C). The TGFb1-added group and JUNB overexpression-alone group showed a weak ability to convert from CD90-negative to CD90-positive, but the addition of TGFB1 to JUNB-overexpressing cells additively increased the conversion rate. Therefore, we investigated the activation of AKT by increased JUNB expression by TGFb1, which exists upstream and downstream of AP1 (Fig. 7C). When recombinant TGFb1 was added to control cells and JUNB overexpressing cells in a concentration-dependent manner, the level of AKT pathway activation was highest with TGFb1 + JUNB treatment. AKT activation by high JUNB expression provides a foundation for promoting transformation to CD90-positive cells, and further promotes the activation of this signaling pathway in the presence of TGFb1. Coculture of fibroblasts treated with a TGFR1 inhibitor for 3 days with epithelial hepatoma cells confirmed that fibroblast-derived paracrine TGFb1 is sufficient for the acquisition of metastatic ability by epithelial hepatoma cells (Figs. 1C, 3B, and S6A–C). Therefore, the phenomenon in which JUNB-positive HCC cells are abundantly present near the fibers surrounding the tumor in pathological specimens of subcutaneous tumors upon mixing epithelial HCC cells with Tig3-20 cells is convincing (Fig. S2A, B).

### Human HCC tissue specimens metastasizing to the lungs contain many JUNB-positive HCC cells near the tumor nodule

**Fig. 8:**
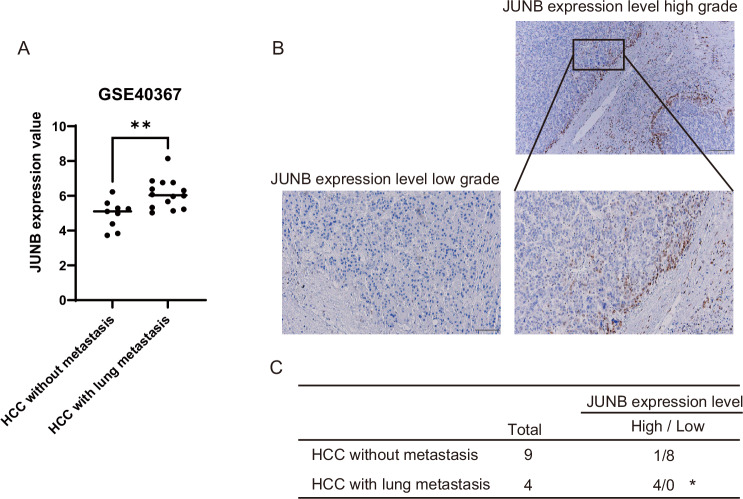
Primary tumors of human HCC with high JUNB expression have a high probability of metastasizing to the lungs. **A** Diagram depicting the comparison of the expression of JUNB in the primary human HCC cases depending on the presence (*n* = 14) or absence (*n* = 11) of metastasis to the lungs (GSE40367). **B** Immunohistochemical staining images showing the localization of JUNB expression in the primary human HCC. **C** Diagram showing comparison of the expression of JUNB determined using immunohistochemical staining of the primary human HCC specimens according to the presence (*N* = 9) or absence (*n* = 4) of lung metastasis. In **A**, data are presented as the mean (SD) and were analyzed using the Mann–Whitney *U*-test. ^*^P < 0.05. In **C**, data were analyzed using the *χ*^*2*^ test. ^*^P < 0.05.

We confirmed that JUNB mRNA levels in clinical specimens of human primary HCC with lung metastasis were higher than those of primary HCC without lung metastasis (Fig. 8A). The localization of JUNB in primary tumors of human HCC with lung metastasis was also similar to that in mouse subcutaneous tumors, with many positive cells near the tumor nodule (Fig. 8B). JUNB-positive HCC cells were present at a high frequency (100%) in 4 of 4 primary HCC samples with lung metastasis, whereas they were almost absent (11%) in 1 of 9 primary HCC samples without lung metastasis (Fig. 8C). These results indicate that high JUNB expression in epithelial HCC cells leads to high lung metastasis, suggesting that JUNB may be a poor prognostic factor in HCC.

## Discussion

In HCC, epithelial CSCs express EpCAM whereas mesenchymal CSCs express CD90. Transformation between these CSCs remains unexplored. In tumor tissue, various stromal cells form a tumor-promoting microenvironment, CSCs, and stromal cells exist adjacent to each other at the tumor front [21]. In this study, we focused on CSCs and fibroblasts, which contribute to HCC, and explored the transformation of CSCs, the transcription factors involved in the transformation, and the signaling pathway. By coculturing HCC EpCAM-positive cells with fibroblasts, we generated CD90-positive cells from EpCAM-positive cells and confirmed that epithelial HCC acquires metastasizing ability. We also identified JUNB as a transcription factor that induces transformation of epithelial EpCAM-positive to mesenchymal CD90-positive cells. HCC cells in which JUNB expression was increased by fibroblast-derived TGFb1 were transformed into mesenchymal cancer cells, with potential to metastasize to the lungs.

JUNB is an AP-1 family transcription factor that forms complexes with Jun, Fos, ATF, and MAF family genes and exerts its physiological functions as does AP-1. AP-1 controls many cellular processes, including cell division, growth, differentiation, migration, and death. Aberrant AP-1 activity promotes invasion and metastasis, enhances angiogenesis, and leads to carcinogenesis [22–24]. As such, JUNB may perform a variety of functions. RNA-seq analysis showed that increased JUNB expression in epithelial HCC cells enhanced the expression of many genes involved in ECM organization, angiogenesis, and regulation of bone remodeling, and this was accompanied by activation of the PI3K-Akt signaling pathway [25]. These results indicated that epithelial HCC cells with increased JUNB expression exhibit enhanced metastasis and tumor malignancy by undergoing an EMT-like change. AP-1 is activated by the TGFb1/Smad pathway [22]. TGFb1 stimulation increased JUNB expression in epithelial HCC cells in vitro. Furthermore, JUNB increased the metastatic potential of epithelial HCC cells by enhancing the activation of PI3K-AKT, a non-canonical TGFb1 pathway. JUNB can bind to 11,928 DNA sites as a transcription factor in metastatic cells. Most of the JUNB-binding sites were located on the promoter regions of genes involved in the apoptotic process, ECM organization, axon guidance, and angiogenesis. The AP1 components of the promoter regions of 38% of genes that may be directly regulated by JUNB are unknown. In the future, we need to identify the components that bind to JUNB and elucidate the mechanism of JUNB-induced metastasis.

Increased JUNB expression efficiently transformed CD90-negative HCC cells into CD90-positive HCC cells and controlled distant metastasis. However, CD90-positive HCC cells obtained by coculture with fibroblasts showed lower JUNB expression than did CD90-negative cells (Fig. S7A, B). CD90-positive HCC cells exhibit high expression of TGFb1-related genes4. When cells with high JUNB expression were stimulated with TGFb1, the conversion rate to CD90-positive cells increased dramatically (Fig. S5A–C). Based on these results, we surmise that JUNB is necessary for transformation to CD90-positive cells but is not necessary for the growth of CD90-positive cells. Moreover, paracrine stimulation of TGFb1 from fibroblasts was more important for transformation to mesenchymal cancer cells than the activation of the TGFb1 signaling in the tumor cells themselves in coculture with fibroblasts. Activation of the PI3K-AKT pathway in HCC cells by paracrine TGFb1 from CAFs was necessary to increase the efficiency of JUNB-induced acquisition of metastatic potential. CD105, c-KIT, VEGFR1, and some other proteins are involved in maintaining the self-renewal and proliferation ability of CD90-positive hepatoma cells [26, 27]. These factors may also increase the rate of JUNB-mediated transformation into CD90-positive hepatoma cells.

SOX4 may also play a role in the transformation of HCC CSCs. SOX4 contributes to EMT [28, 29]. The expression of SOX4 was markedly increased in the co-cultures of CD90-negative cells and fibroblasts Tig3-20. SOX4 is also involved in the transformation of HCC CSCs. However, SOX4 was not involved in the acquisition of metastatic potential via TGFb1 for CD90-negative HCCs. Thus, there may be a mechanism for the acquisition of metastatic potential via SOX4 by fibroblast-derived cytokines other than TGFb1.

Patients with JUNB-positive HCCs near tumor nodules have a high potential for lung metastasis. This phenomenon is caused by the transition of epithelial liver CSCs into CD90-positive HCC cells. Therefore, inhibiting JUNB in primary tumors may be effective in suppressing distant metastasis. However, the mechanism of transformation into CD90-positive cells, which is directly controlled by JUNB, needs to be investigated in detail. In addition, because JUNB expression in epithelial HCCs is related to fibrosis, apoptosis, and angiogenesis in addition to cancer metastasis, it might be involved in the immune environment in tumor tissue. Combination therapy with a JUNB inhibitor and immune checkpoint inhibitors may be effective as a new treatment for HCC.

## Materials and methods

### Reagents

Human TGFb1 recombinant protein was purchased from Sigma-Aldrich Japan K.K. (T7039, Tokyo, Japan). TGF-β RI kinase inhibitor was obtained from Merck Millipore (CAS396129-53-6, Darmstadt, Germany).

### Clinical samples

Pathological specimens were obtained with informed consent from patients who underwent liver cancer resection at Kanazawa University Hospital Liver Disease Center from 2010 to 2017. Pathological slides were prepared using samples from four primary cancer cases with lung metastasis and nine primary cancer cases without lung metastasis and were analyzed immunohistologically. Ethics approval was provided by the Institutional Review Board of the Graduate School of Medical Science, Kanazawa University (2017-323).

### Cell culture

Huh7, Huh1, and TIG3-20 cells were purchased from the Japanese Collection of Research Bioresources Cell Bank (Osaka, Japan), and human HSC (Lx-2) were obtained from Dr. Scott Friedman (Mount Sinai School of Medicine, New York, NY). Huh7, Huh1, Lx2, and TIG3-20 lines were maintained in Dulbecco’s modified Eagle medium (ThermoFisher Scientific, Waltham, MA, USA) supplemented with 10% fetal bovine serum, 100 U/mL penicillin, and 100 μg/mL streptomycin.

Fresh human HCC surgical specimens were disaggregated into cell suspensions using gentleMACS™ Dissociators following the protocol provided with the Tumor Dissociation Kit, human (130-095-929, Miltenyi Biotec, Tokyo, Japan). Red blood cells in the cell suspension were hemolyzed with ammonium chloride solution (Stemcell Technologies, Inc., Vancouver, Canada) on ice for 5 min. Single cell suspensions were inoculated into subcutaneous lesions of NOD/SCID mice. The subcutaneous tumors that were formed were dissected, digested, and subsequently used for in vitro cell culture. The human HCCs used in this study were the same cells as HCC1 in *Cancer Cell International volume 17, Article number: 94 (2017)*, which has been confirmed by whole exosome sequence analysis, and *Hepatology 57(4):p 1484–1497, April 2013*. The HCC of P7 is the patient's HCC used in this study. How the cell line was created was detailed, and its tumorigenic potential and CSCs were analyzed.

### Creation of GFP-labeled human liver cancer cell line and Human SOX4- and JUNB-overexpressing cell lines

The open-reading frame (ORF) of EGFP was amplified using PCR with the following primers: Forward: 5′-TAACTCGAGGCCACCATGGTGAGCAAGGGCGAGGAGCT-3′; Reverse: 5′-AATGGATCCTCACTTGTACAGCTCGTCCATGCCGA-3′. Plasmids harboring human JUNB (NM_002229.3) and SOX4 (NM_003107.3) ORFs were purchased from GenScript (Tokyo, Japan). SOX4 was amplified using the following primers: Forward: 5′-TAACTCGAGGCCGCCATGGTGCAGCAAACCAAAATGCGA-3′; Reverse: 5′-AATGGATCCTCACTTGTCATCGTCATCCTTGTAGTCGTAGGTGAAAACCAGGTTGGAGA-3′. JUNB was amplified using the following primers: 5′-TAACTCGAGGCCGCCATGTGCACTAAAATGGAACAGCCCTT-3′; Reverse: 5′- AATGGATCCTCACTTGTCATCGTCATCCTTGTAGTCGAAGGCGTGTCCCTTGACCCCAA-3′. The amplified PCR fragment was treated with XhoI/BamH1. This DNA fragment was mixed with pLVSIN-CMV Pur Vector (TAKARA), treated with XhoI/BamH1, and ligated using a Ligation Mix.

Lentiviral particles were produced by transfecting genes into the Lenti-X™ 293T cell line according to the protocol provided with the pLVSIN puro vector and Lentiviral High Titer Packaging Mix (TAKARA). Huh7, Huh1, and patient HCC cell lines were infected with the recombinant lentivirus and selected using 2 mg/mL puromycin.

### RNA extraction, reverse transcription, and real-time PCR

Total RNA was extracted from the cell lines using the RNeasy Mini Kit (Qiagen, Valencia, CA, USA). cDNA was synthesized using the High-Capacity cDNA Reverse Transcription Kit (ThermoFisher Scientific), following the manufacturer’s protocol. Gene expression analysis was performed using human TaqMan probes for *THY1* (Hs00174816_m1), *JUNB* (Hs00357891_s1), *SOX4* (Hs04987498_s1), *TGGB1* (Hs00171257_m1), *TWIST2* (Hs02379973_m1), *ZEB1* (Hs00232783_m1), *ZEB2* (00207691_m1), *SNAI1* (Hs00195591_m1), *SNAI2* (Hs00950344_m1), and *GAPDH* (Hs99999903_m1) on a QuantStudio 12 K Flex real-time PCR system (ThermoFisher Scientific).

### Cell proliferation assay

Single-cell suspensions of 2.0 × 10^3^ cells were seeded in the wells of 96-well plates and cultured for 24 h, 48 h, or 72 h. Cell density was evaluated using the Cell Counting Kit-8 (Dojindo Laboratories, Kumamoto, Japan).

### Immunohistochemistry

Immunohistochemistry was performed using EnVision+ Kits (Dako, Carpinteria, CA), according to the manufacturer’s instructions. FFPE tumor tissues were deparaffinized, rehydrated, and subjected to antigen retrieval and protein blocking (Protein Block Serum Free; Dako, Carpinteria, CA, USA); subsequently, the sample slides were incubated overnight at 4 °C with the primary anti-Human JUNB rabbit monoclonal antibody (#3753S, Cell Signaling Technology, Inc, Danvers, MA, USA). IHC images were acquired using a BIOREVO BZ-X810 microscope (Keyence, Osaka, Japan). Sirius Red staining was performed using the Picrosirius Red Stain Kit (24901-250, Polysciences, Inc., Warrington, PA, USA) according to the manufacturer’s protocol.

### Flow cytometry analysis and cell sorting

Cultured cells were trypsinized, washed, and resuspended in PBS supplemented with 1% HEPES and 2% fetal bovine serum. Cells were then incubated with antibodies on ice for 30 min. CD326 (EPCAM)-APC (130-113-260, Miltenyi Biotec) and CD90 (THY1)-APC (17-0909-42, eBioscienceTM) were the fluorescent antibodies used. Determination of the presence or absence of positive cells and the extraction of target cells were performed using BD FACSMelody^TM^ (BD Biosciences, San Jose, CA).

### Immunofluorescence staining

For immunofluorescence staining, anti-CD90 rabbit monoclonal antibody (#130801S, Cell Signaling Technology, Inc.) was incubated with Alexa 594 fluorescein isothiocyanate–conjugated anti-rabbit IgG on ice for 30 min. All images were obtained using a BIOREVO BZ-X810 microscope (Keyence).

### Animal studies

Five-week-old male NOD/SCID mice were purchased from Charles River Laboratories, Inc. (Wilmington, MA, USA). Tumor cells (1 × 10^6^ cells/mice) and Lx2 or TIG3-20 (2 × 10^6^ cells/mice) were mixed in 100 μL of PBS. The mixed cells were suspended in 100 μL of Dulbecco’s modified Eagle medium and Matrigel (Corning, New York, NY) and subcutaneously transplanted into NOD/SCID mice. A JUNB-overexpressing liver cancer cell line was also adjusted to 1 × 10^6^ cells/mouse for experiments. The tumor diameter was measured twice a week from the time of subcutaneous tumor formation, and the progress was observed up to 45 days. Tumor and lung tissues were pathologically evaluated after 45 days. Alternatively, analysis was performed when body weight loss was 30% or more or tumor volume was approximately 10,000 mm^3^.

The study protocol was approved by the Kanazawa University Animal Care and Use Committee. All procedures were performed in accordance with the guidelines and regulations of Kanazawa University.

### Immunoblotting

Protein extracts were prepared by lysing mouse liver tissue in RIPA Lysis Buffer (Merck) containing Protease Inhibitor Cocktail and Phosphatase Inhibitor Tablets (Roche Applied Science, Pleasanton, CA). The primary antibodies used were anti-JUNB rabbit monoclonal antibody (#3753S, Cell Signaling Technology), anti-total AKT rabbit polyclonal antibody (#9272S), anti-pAKT (Ser473) rabbit monoclonal antibody (#4060S), anti-total SMAD3 rabbit polyclonal antibody (#9513S), anti-pSMAD3 (Ser423/425) rabbit polyclonal antibody (#9520S), anti-total SAPK/JNK rabbit polyclonal antibody (#9252S), anti-pSA’K/JNK (Thr183/Tyr185) rabbit polyclonal antibody (#9251S), anti-SOX4 mouse monoclonal antibody (sc-518016, SantaCruz), and anti-GAPDH mouse monoclonal antibody (sc-32233, SantaCruz).

### Original full-length western blots

Original full-length western blots obtained in this study are provided in Supplementary Table 3.

### Whole RNA-seq and ChIP-seq analysis

GFP-labeled cells (5 × 10^5^) were extracted from tumor cells cocultured with fibroblasts and lysed for RNA extraction. The RNeasy Mini Kit (Qiagen, Valencia, CA, USA) was used to extract total RNA from sorted cell lysate. Sequence analysis of each RNA was performed using Illumina NovaSeq 6000. Differential gene expression analysis and chart generation with *P* < .005 by unpaired *T*-test were analyzed by Rhelixa (Tokyo, Japan). RNA-seq data of HCC samples with and without lung metastasis were obtained from Gene Expression Omnibus 40367.

### Statistical analysis

Experimental data were analyzed using GraphPad Prism 10 (GraphPad Software, San Diego, CA, USA). In vitro experimental results were performed using one-way ANOVA. For the in vivo experimental results, *P*-values were determined using the Mann–Whitney *U*-test and *χ*^*2*^ test. Statistical significance was considered at *P* < 0.05.

## Supplementary information

Supplementary Figure Legends

Figure S1

Figure S2

Figure S3

Figure S4

Figure S5

Figure S6

Figure S7

Supplementary Table S1

Supplementary Table S2

Original western blots

## Data Availability

The data supporting the findings of this study are available from the corresponding authors upon request.
