# Supplementary material for: Transcription factor JUNB is required for transformation of EpCAM-positive hepatocellular carcinoma (HCC) cells into CD90-positive HCC cells in vitro
Source: Cell Death Dis. 2025 Apr 19;16(1):319. doi: 10.1038/s41419-025-07602-3 (PMC12009367; doi:10.1038/s41419-025-07602-3)
Supplement: Supplementary file 1 — Supplementary Figure Legends [file 41419_2025_7602_MOESM1_ESM.docx]

**Transcription factor JUNB is required for transformation of EpCAM-positive hepatocellular carcinoma (HCC) cells into CD90-positive HCC cells in vitro**

**Supplementary Figure Legends**

**Supplement Figures:**

**Fig. S1 Changes in the expression of the epithelial HCC cell marker EpCAM on the cell membrane with or without coculture with Lx-2 and Tig3-20 fibroblasts.** A–C Expression of the epithelial marker EpCAM after coculture of GFP-labeled Huh1 (A), Huh7 (B), and patient HCC (C) with Lx-2 and Tig3-20 fibroblasts.

**Fig. S2 Localization of JUNB expression in subcutaneous tumors created by subcutaneously implanting a mixture of epithelial liver cancer cell lines (Huh1 or Huh7) and Tig3-20 fibroblast cell line.** A, B Images of immunohistochemical staining of JUNB in subcutaneous tumors of epithelial liver cancer cells Huh1 (A) and Huh7 (B) compared with and without mixture of Tig3-20 fibroblasts.

**Fig. S3. Reduction in the proliferation of human epithelial HCC cells overexpressing JUNB compared with that of the control.** A–C Graph of MTS assay for evaluating the time-dependent proliferation of JUNB-overexpressing epithelial HCC cells Huh1 (A), Huh7 (B), and patient HCC (C). Data are presented as the mean (SD) (n = 3) and were analyzed using Student’s t-test. ***P < 0.001.

**Fig. S4 Overexpression of JUNB in epithelial HCC cells increases the expression of EMT-related genes.** A–C mRNA levels of TGFb1, TWIST2, ZEB1, ZEB2, SNAIL1, and SLUG in three types of JUNB-overexpressing epithelial HCC cells: Huh1 (A), Huh7 (B), and patient HCC (C). Data are presented as the mean (SD) (n = 3) and were analyzed using the Student’s t-test. *P < 0.05, **P < 0.01, ***P < 0.001.

**Fig. S5 Increase in the rate of conversion to CD90-positive cells when epithelial liver cancer cells overexpressing JUNB were stimulated with TGFb1.** A–C Flow cytometry analysis of CD90 antibody bound to APC after culturing epithelial hepatoma cells Huh1 (A), Huh7 (B), and patient HCC (C) for 72 h without treatment, with TGFb1, JUNB overexpression, or with JUNB overexpression + TGFb1.

**Fig. S6 Transformation of EpCAM-positive HCC cells into CD90-positive cells is dependent on the paracrine effect of fibroblast-derived TGFb1.** A Diagram of three types of experimental schedules shown as I–III. In I, EpCAM-positive epithelial HCC cells were treated with TGFb1 RI inhibitor and cultured for 24 h. In II, EpCAM-positive epithelial HCC cells were treated with TGFb1 RI inhibitor and cultured for 24 h, mixed with Tig3-20 fibroblasts, and cultured for 72 h. In III, Tig3-20 fibroblasts were treated with TGFb1 RI inhibitor and cultured for 24 h, and then mixed with EpCAM-positive epithelial HCC cells and cultured for 72 h. B–D Diagram showing the evaluation of the CD90-positivity rate using flow cytometry of EpCAM-positive epithelial HCC cells Huh1 (B), Huh7 (C), and patient HCC (D), carried out according to the experimental schedule shown in (A).

**Fig. S7 CD90-positive HCC cells generated by coculture with Tig3-20 fibroblasts showing lower JUNB expression than that in CD90-negative HCC cells.** A, B EpCAM-positive HCC cells Huh1, Huh7, and patient HCC were cocultured with Tig3-20 fibroblasts for 72 h. Expression levels of SOX4 and JUNB mRNA in CD90-positive and -negative cells after culture are shown. Data are presented as the mean (SD) (n = 3) and were analyzed using Student’s t-test. *P < 0.05, **P < 0.01, ***P < 0.001.
