# Supplementary figures and images for: Transcription factor JUNB is required for transformation of EpCAM-positive hepatocellular carcinoma (HCC) cells into CD90-positive HCC cells in vitro
Source: Cell Death Dis. 2025 Apr 19;16(1):319. doi: 10.1038/s41419-025-07602-3 (PMC12009367; doi:10.1038/s41419-025-07602-3)

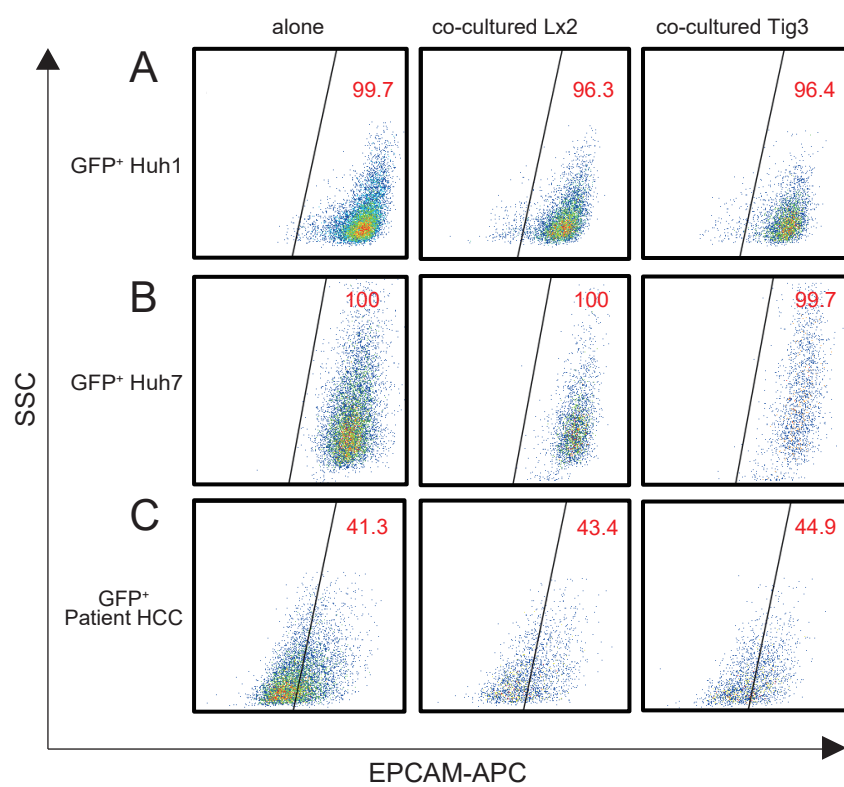

Supplement: Supplementary file 2 — Figure S1 [file 41419_2025_7602_MOESM2_ESM.pdf]

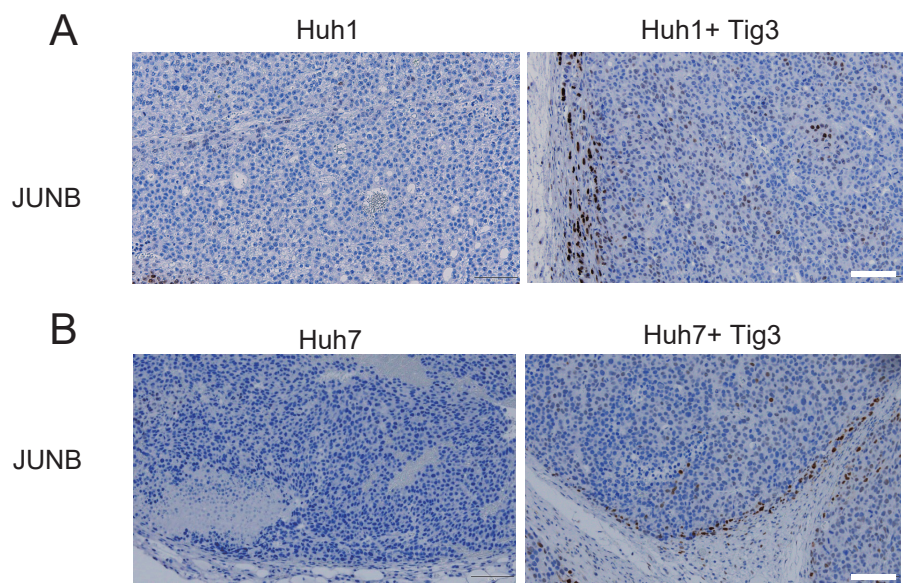

Supplement: Supplementary file 3 — Figure S2 [file 41419_2025_7602_MOESM3_ESM.pdf]

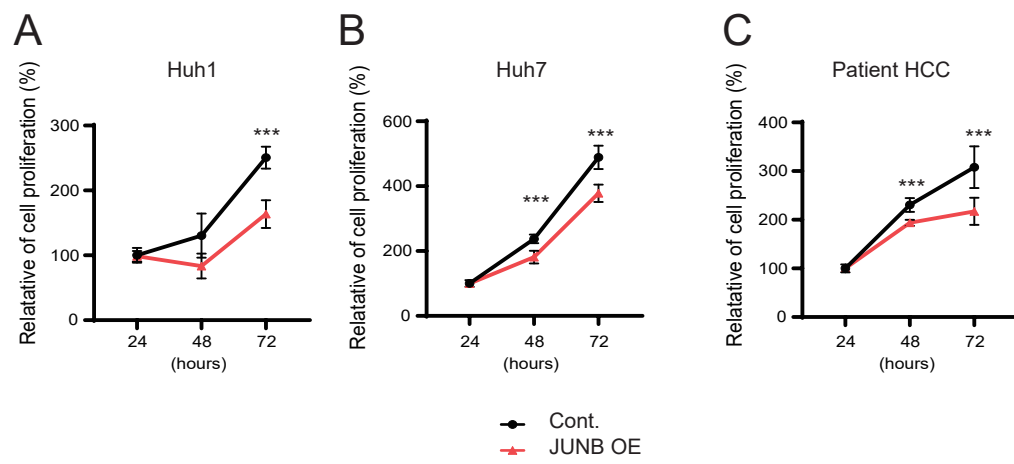

Supplement: Supplementary file 4 — Figure S3 [file 41419_2025_7602_MOESM4_ESM.pdf]

A

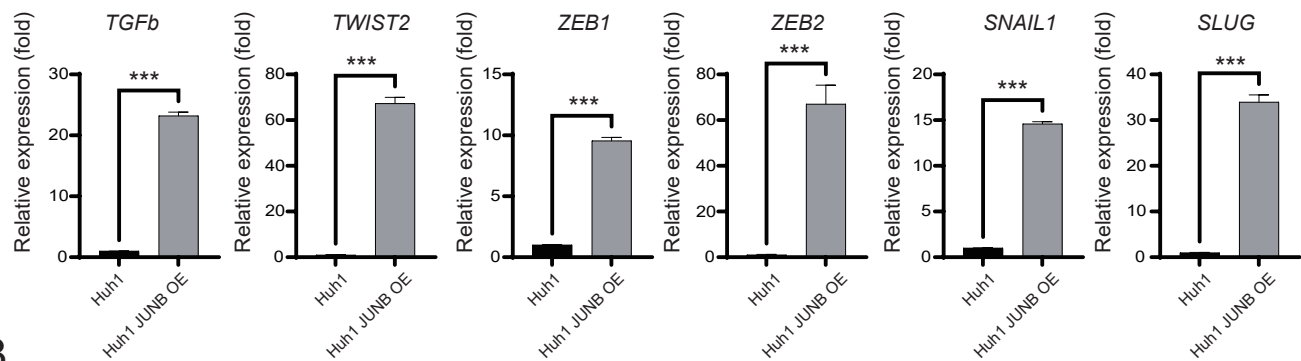

B

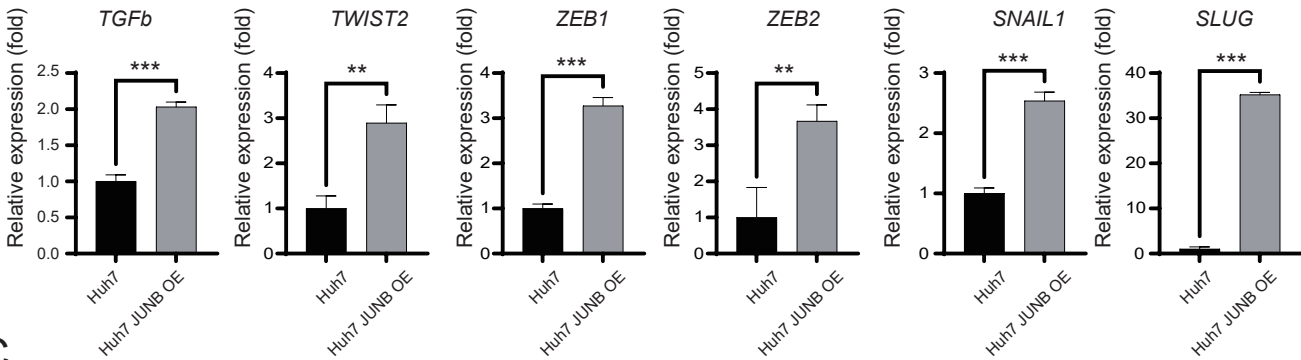

C

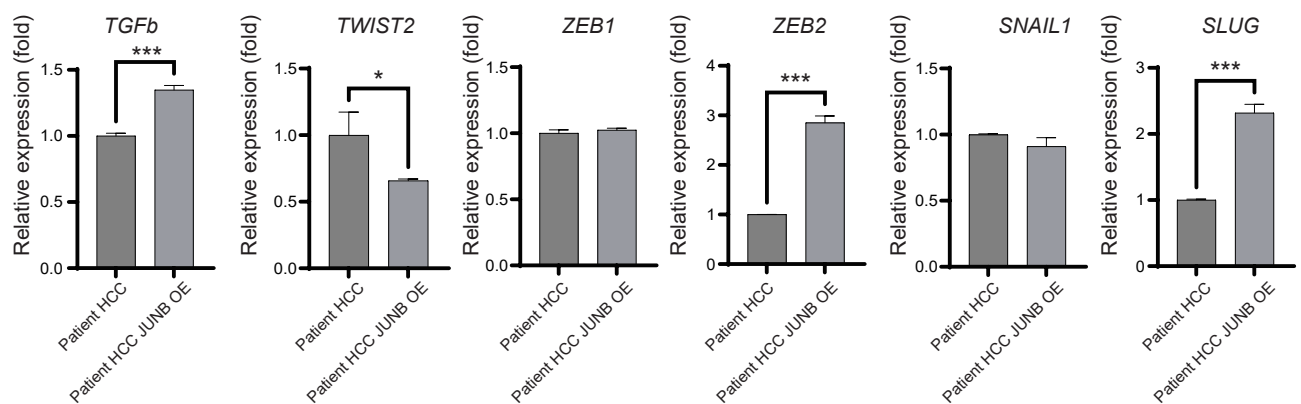

Supplement: Supplementary file 5 — Figure S4 [file 41419_2025_7602_MOESM5_ESM.pdf]

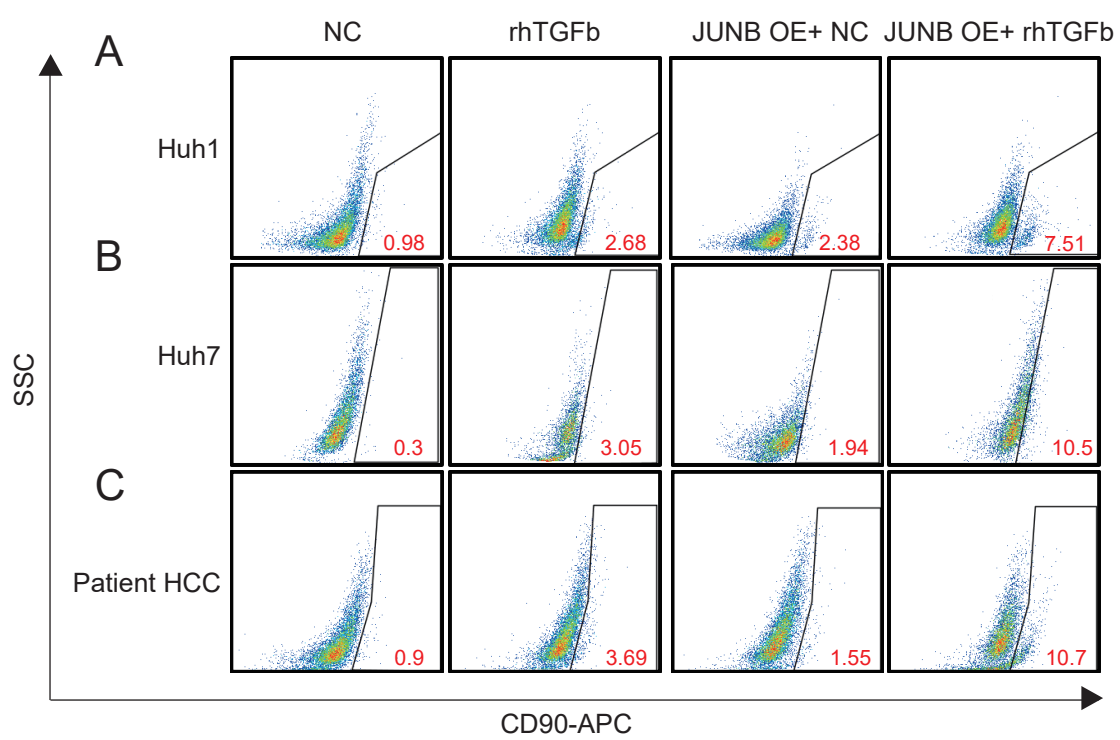

Supplement: Supplementary file 6 — Figure S5 [file 41419_2025_7602_MOESM6_ESM.pdf]

A

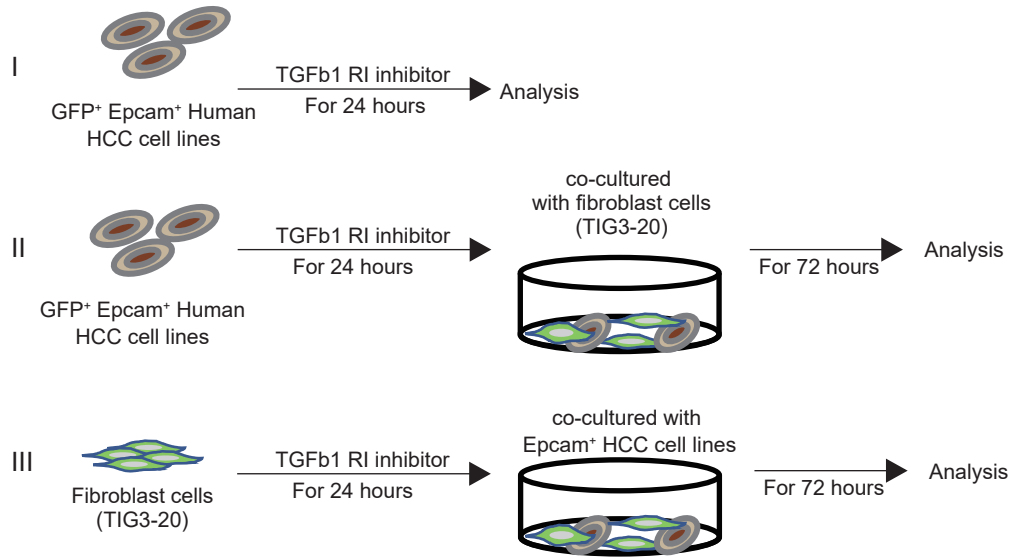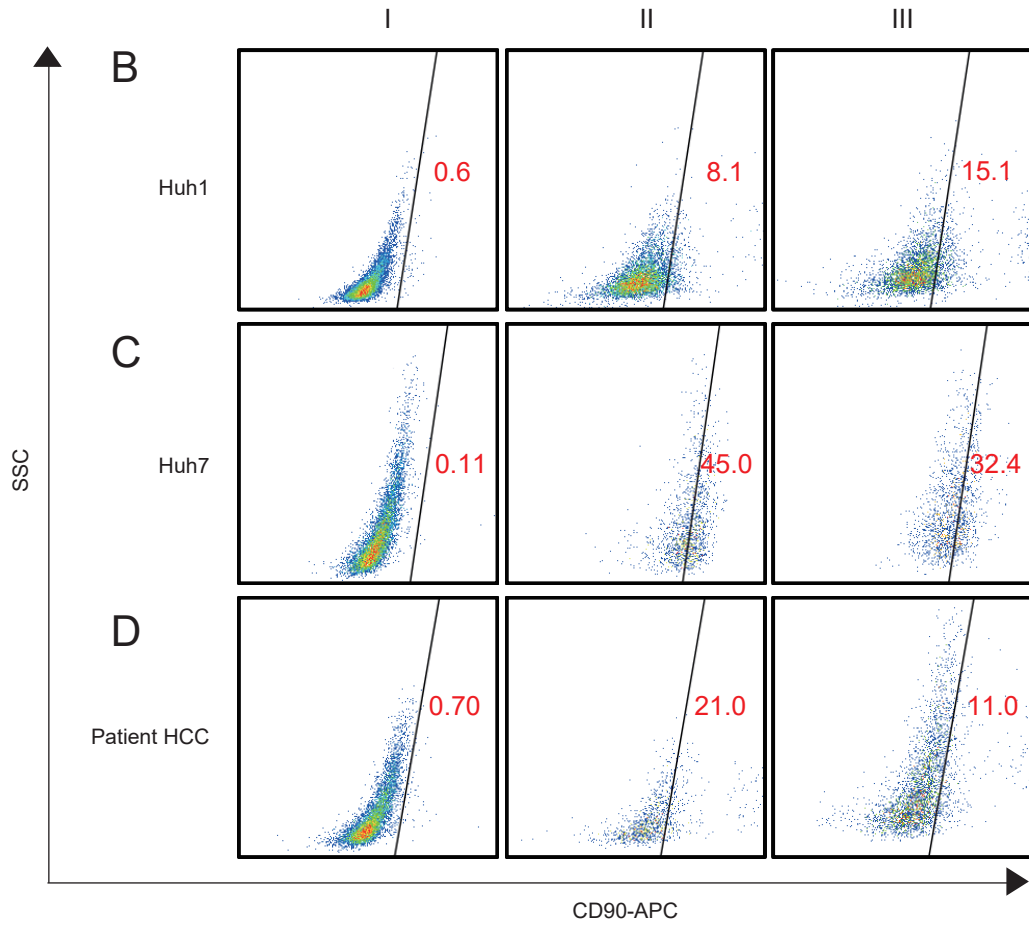

Supplement: Supplementary file 7 — Figure S6 [file 41419_2025_7602_MOESM7_ESM.pdf]

A

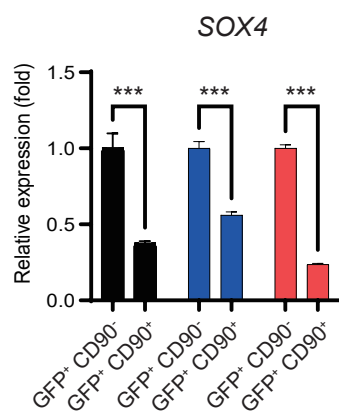

B

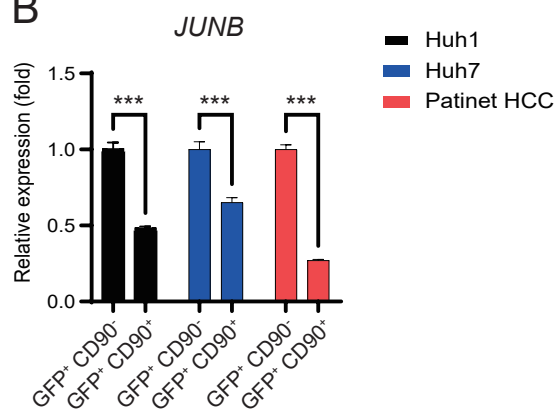

Supplement: Supplementary file 8 — Figure S7 [file 41419_2025_7602_MOESM8_ESM.pdf]

Supplementary Table S3

Figure3H

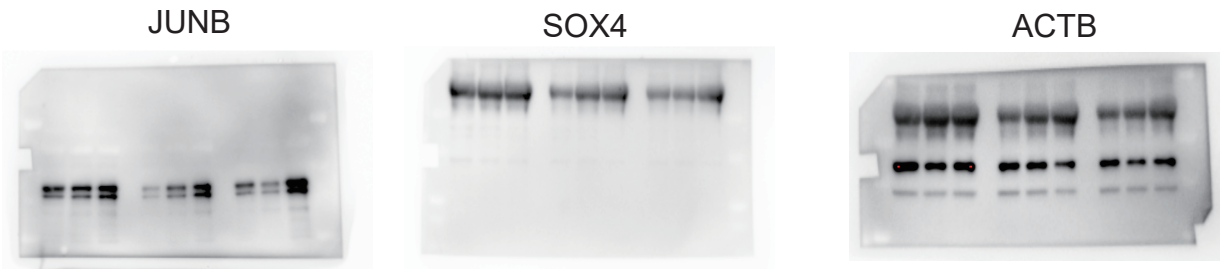

Figure4A

Figure4B

Figure4C

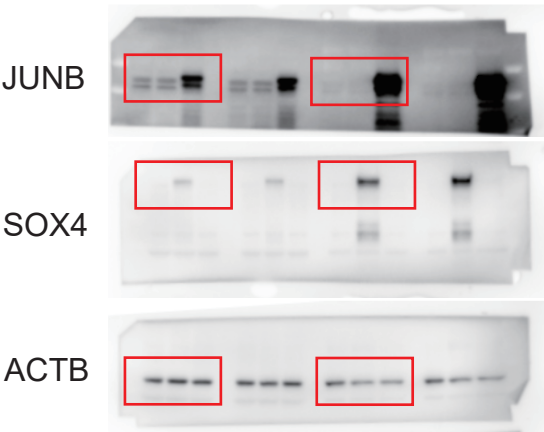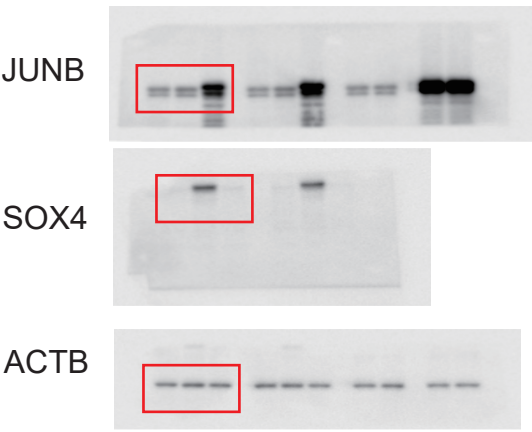

Figure 7B

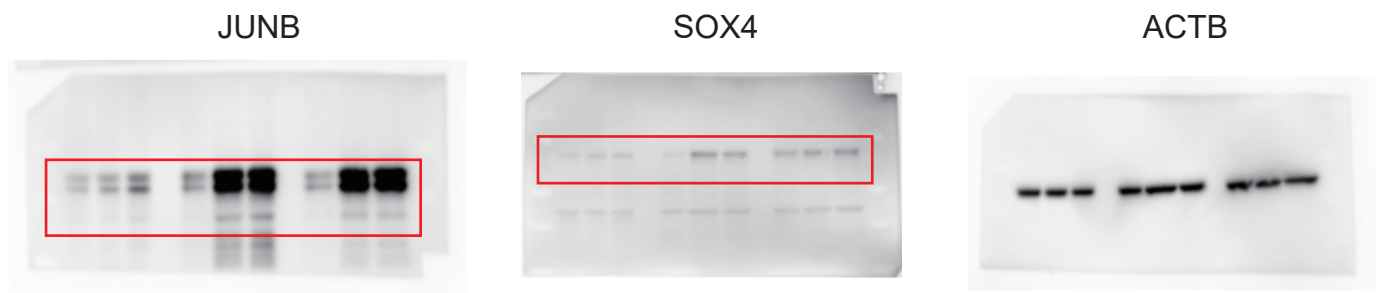

Figure 7C

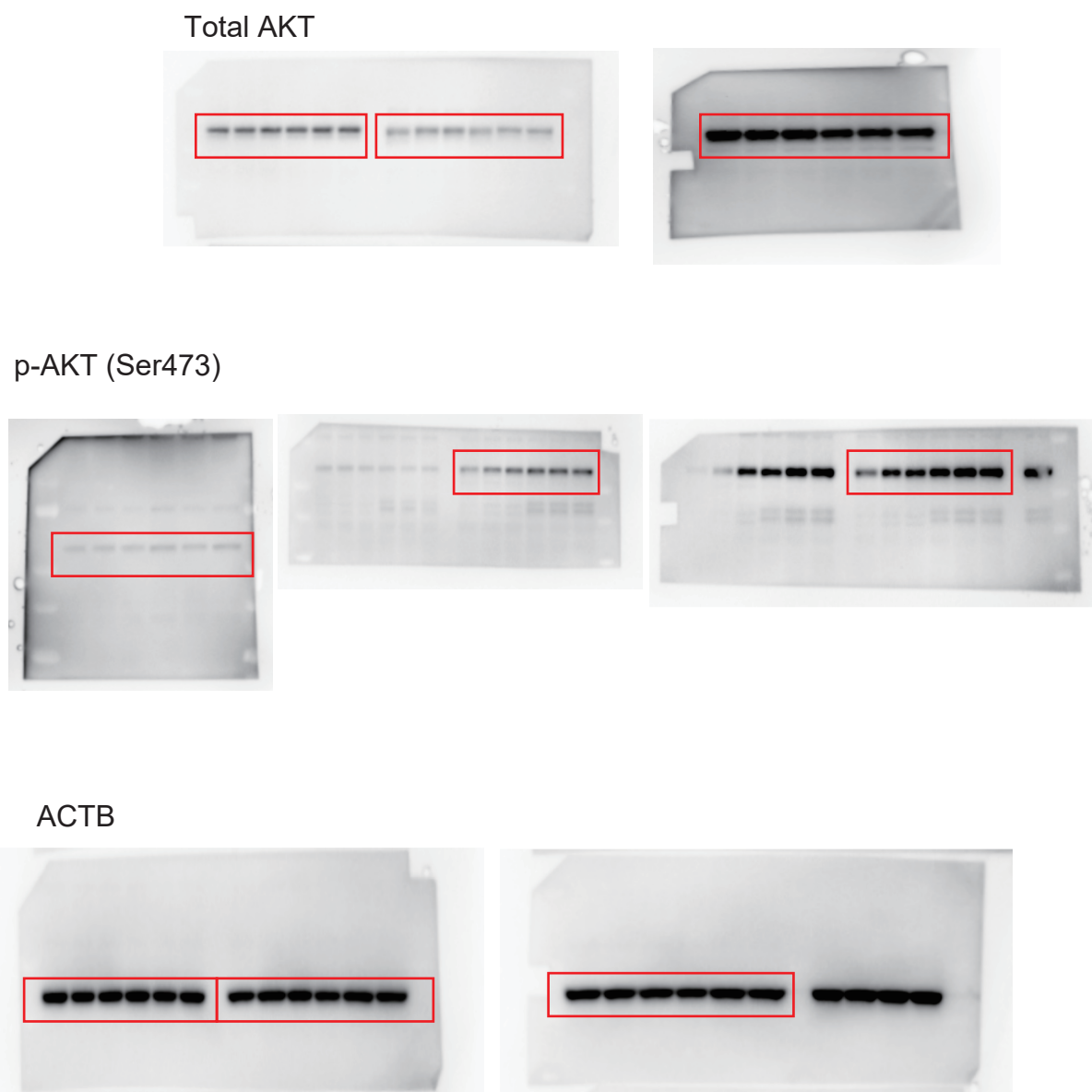

Supplement: Supplementary file 11 — Original western blots [file 41419_2025_7602_MOESM11_ESM.pdf]
